# Greater Cochlear Damage in Otogenic Versus Meningogenic Meningitis: Hearing Rehabilitation Implications

**DOI:** 10.1002/lary.70555

**Published:** 2026-04-08

**Authors:** Matheus Pedrosa Tavares, Henrique Furlan Pauna, Rafael da Costa Monsanto, Nevra Keskin‐Yilmaz, Breno Lima de Almeida, Norma de Oliveira Penido

**Affiliations:** ^1^ Universidade Federal de São Paulo São Paulo São Paulo Brazil; ^2^ Pontifícia Universidade Católica do Paraná, Curitiba‐PR, Brazil Curitiba Paraná Brazil; ^3^ University of Minnesota Minneapolis Minnesota USA

**Keywords:** hearing loss, histology, labyrinthitis, meningitis, otopathology, temporal bone

## Abstract

**Objective:**

To quantify cochlear and vestibular cellular losses between cases of meningogenic and otogenic meningitis.

**Methods:**

From the archival human temporal bone collection at the University of Minnesota, we selected specimens with meningitis history and histopathological evidence of labyrinthitis. We grouped specimens into two categories (otogenic and meningogenic) based on infection route and included age‐matched controls without ear or central nervous system disease. From 36 temporal bones, we quantitatively assessed outer hair cell (OHC) and inner hair cell (IHC) loss, as well as spiral ganglion neuron (SGN) and Scarpa's ganglion neuron (ScGN) counts, then compared results among groups.

**Results:**

Both case groups demonstrated OHC loss compared with controls (*p* < 0.05), with more severe loss in the otogenic group versus the meningogenic group (*p* = 0.01). IHC loss occurred only in the otogenic group compared with both meningogenic and control groups (*p* = 0.019 and < 0.001, respectively). No statistically significant difference was found between IHC loss in meningogenic and control groups (*p* = 0.382). Both otogenic and meningogenic groups showed significant reduction of SGN and ScGN counts compared with controls (*p* < 0.05), with no statistically significant differences between the two meningitis groups for either measure (*p* = 0.993 and 0.762, respectively).

**Conclusion:**

Meningitis is associated with loss of cochlear hair cells, SGN and ScGN. The otogenic route demonstrated a greater loss of both IHC and OHC in comparison with the meningogenic route.

**Level of Evidence:**

N/A.

## Graphical abstract

Otogenic route of meningitis was associated with greater cochlear hair cell loss in comparison with the meningogenic one in this otopathology study. Neuronal depletion has been traditionally implicated in failed hearing rehabilitation of post‐meningitis deafness. This histopathological finding provides a basis for investigating additional factors that may influence these suboptimal results.

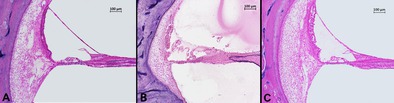

## 1 Introduction

Meningitis is defined as the inflammation of the coverings of the brain or spinal cord. It remains a major global public health concern as morbidity and mortality from meningitis remain high. There were an estimated 2.51 million cases of meningitis worldwide in 2019 [1]. Approximately 20% of individuals affected with meningitis develop long‐term complications, and hearing loss (HL) is the most common one [2]. There is a classic triad of clinical presentation which consists of fever, neck stiffness, and altered mental status or headache. Constitutional symptoms, seizures, focal neurological deficits, and cranial nerve palsies can also be present [3]. Meningitis can be caused by either bacteria or virus. The most common cause of bacterial meningitis is *Streptococcus pneumoniae* , but *Neisseria meningitidis* and *Haemophilus influenzae* are other important causes [1]. It is particularly prevalent among individuals with contiguous infections, such as otitis media (OM), mastoiditis, and sinusitis [4]. Empiric antimicrobial treatment is indicated when bacterial meningitis is suspected, and early administration of systemic corticosteroids is indicated to reduce the risk of death and neurological complications, including HL, especially in pneumococcal and *H. influenzae* meningitis [5, 6].

Meningitis is a known potential complication of OM. *S. pneumoniae* is also the most common bacterial cause of acute OM. Otogenic meningitis is defined as the presence of concurrent OM in a case of meningitis. It is found in 31% of the cases [7]. The otogenic route, in which OM can access the central nervous system, occurs via direct and indirect pathways. The first includes normal anatomical pathways (the round and oval windows, modiolus, cochlear aqueduct, internal auditory canal) or areas of bone erosion [8, 9, 10, 11]. The latter includes haematogenous spread due to systemic bacteraemia or thrombosis of the emissary veins of the dura [12]. The concurrent OM can be identified through clinical presentation of otologic symptoms, alterations in otoscopy, imaging tests findings, or postmortem histopathology findings. In the cases of meningogenic route, the access to the inner ear is through cochlear aqueduct from the infected subarachnoid space, during the first days, and least likely via hematogenous invasion via the spiral ligament at later stages, as demonstrated in animal model studies [13, 14].

The prevalence of HL in pneumococcal meningitis ranges between 20% and 26%, and the prevalence of bilateral HL of at least 70 dB is 8% [15]. HL can occur at admission or during the disease course. Transient cases are usually associated with conductive disorders, and permanent HL is generally caused by the involvement of the eighth cranial nerve, cochlea, and labyrinth [15]. Labyrinthitis is the inflammation of the inner ear, and labyrinthitis ossificans is a process of ossification of the inner ear, which usually occurs after the first one. The latter is a known cause of permanent HL and of complicated cochlear implant electrode insertion. However, even cases without cochlear ossification may develop permanent hearing impairment requiring rehabilitation. Formal audiological screening tests should be performed as the goal is to minimize the time to initiation of early hearing rehabilitation. Unnecessary delays may increase the risk of rehabilitation failure, particularly due to cochlear ossification and its potential impact on cochlear implant feasibility [16]. Some studies have reported poorer rehabilitation outcomes after cochlear implant in some cases of post‐meningitis deafness even in the absence of cochlear ossification [17, 18, 19].

No previous study has evaluated the difference in loss of cochlear and vestibular hair cells in cases of otogenic versus meningogenic meningitis. A better understanding of the severity and patterns of cochlear and vestibular degeneration will lay the necessary groundwork for future development of future pathology‐oriented prevention, diagnostic, and treatment strategies. In a previous publication of our laboratory, we performed a comprehensive structural investigation of patterns of cochlear and vestibular lesions which indicated that otogenic cases may result in more severe cochlear damage as compared with meningogenic cases [20]. Therefore, in the present study, we aim to further investigate and quantify cochlear and vestibular cellular losses between cases of otogenic and meningogenic meningitis.

## 2 Materials and Methods

From the archival temporal bone (TB) collection at the Paparella Otopathology & Pathogenesis Laboratory, University of Minnesota, we selected TBs from cases with a history of meningitis. Human TB studies utilizing our archival collection are Institutional Review Board‐exempt (ID: STUDY00003249). All of the archival TBs had been removed at autopsy and processed using a standard protocol [21]. All TBs were fixed in 10% buffered formalin, decalcified with ethylenediaminetetraacetic or trichloroacetic acid, dehydrated in graded concentrations of alcohol, and embedded in celloidin. Sections in the horizontal plane at a thickness of 20 μm were made, and every 10th section was stained using hematoxylin–eosin.

Inclusion criteria were a documented history of meningitis and histopathological evidence of labyrinthitis. Exclusion criteria were (1) pre‐existing auditory or vestibular dysfunction prior to meningitis, such as cochlear otosclerosis, but only if there was endosteal involvement, and Meniere's disease, (2) a history of middle or inner ear surgery, such as mastoidectomy or cochlear implantation, (3) a history of metastatic disease, (4) a history of chemotherapy or radiotherapy, and (5) TBs exhibiting severe postmortem changes.

Specimens were grouped into two categories (otogenic and meningogenic) based on the route of infection: (1) otogenic: medical records indicating middle ear infection as the etiology of meningitis and/or those demonstrating histopathological evidence of acute or chronic middle ear inflammation (such as purulent material, mucosal thickening, granulation tissue or cholesteatoma) and (2) meningogenic: cases lacking a medical history of OM and devoid of any pathological middle ear findings were classified as meningogenic labyrinthitis. We also included a third group as control, which were TBs of individuals without medical history or histopathological evidence of ear or central nervous system disease. All patient data were deidentified.

For each TB, five mid‐modiolar sections were selected for analysis. The central mid‐modiolar section was identified as the slide with maximum modiolar cross‐sectional area and complete or near‐complete visualization of all cochlear turns. Two sections superior and two sections inferior to this central reference section were then selected at equal intervals from the available mounted slides (every 10th section, 20 μm thickness). To count the cells, we used a light microscope (Nikon Eclipse E100; Nikon Co., Tokyo, Japan) with a high‐resolution camera attached (Nikon DS‐Fi3; Nikon Co., Tokyo, Japan). The images were captured at a ×400 magnification and then transferred to an image viewer software (NIS elements viewer—Nikon Co.; Tokyo, Japan).

**FIGURE 1:**
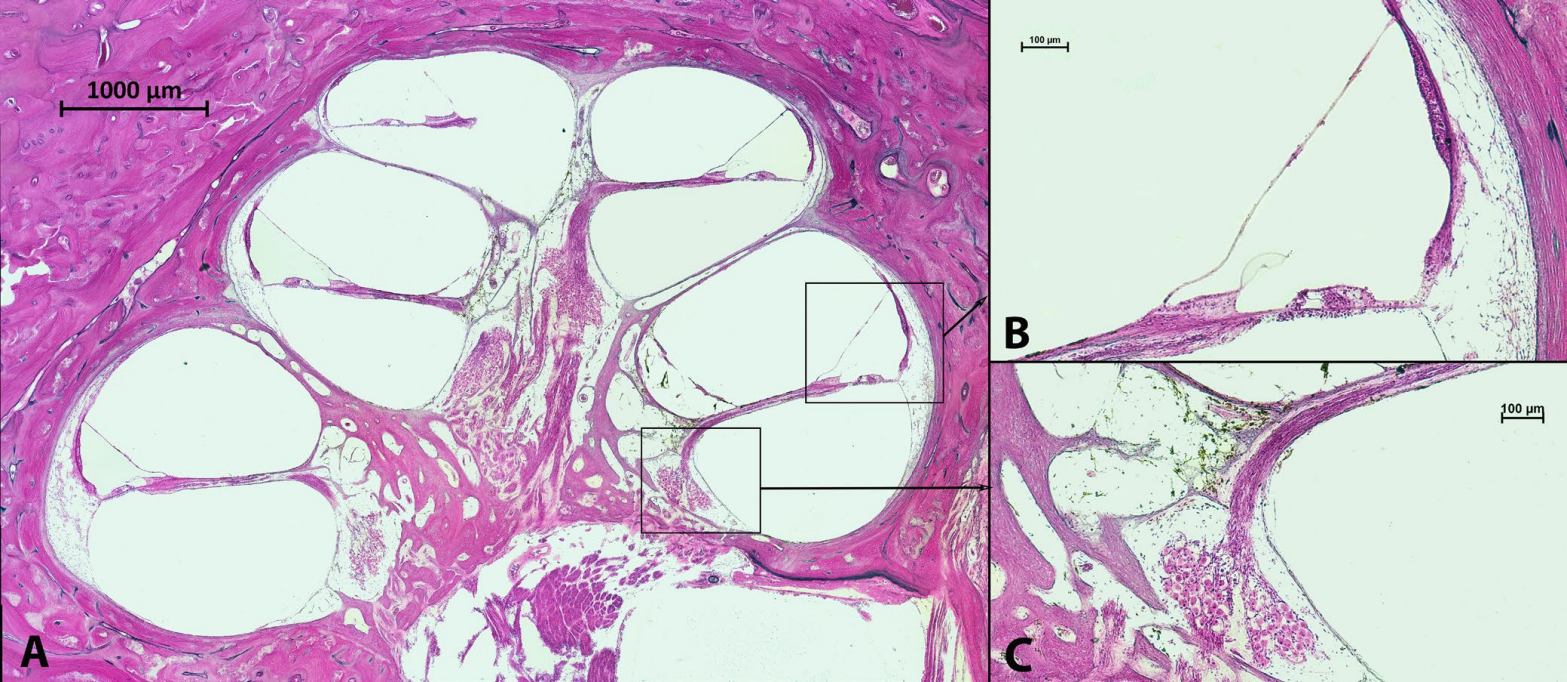
(A) Representation of a normal cochlea (hematoxylin–eosin, original magnification ×4). (B) Enlarged image of the superior framed area in (A) demonstrating organ of corti and cochlear hair cells (hematoxylin–eosin, original magnification ×10). (C) Enlarged image of the inferior framed area in (A) demonstrating spiral ganglion neurons (hematoxylin–eosin, original magnification ×10).

To quantify cochlear hair cell loss, we evaluated each Organ of Corti, as represented in Figure 1, across all cochlear turns—lower basal, upper basal, lower middle, upper middle, and apical turns. Cochlear hair cells were identified by visualizing cell nuclei and cuticular plates. For each organ of Corti in each cochlear turn, inner hair cells (IHCs) were scored as present (if any IHCs were visible) or absent (if no IHCs were identifiable). IHC loss was calculated as the percentage of organs of Corti with absent IHCs across all examined sections and turns. Outer hair cell (OHC) loss was quantified as the number of missing OHCs divided by the expected total count—three OHCs expected per Organ of Corti; therefore, 15 OHCs per mid‐modiolar section across the five cochlear turns. For hair cell quantification, images were obtained at the focal plane in which the nuclei of the hair cells were most clearly visualized. This approach was applied consistently across all specimens to ensure reproducibility and to minimize variability related to section thickness. Because the sections were 20 μm thick, care was taken to avoid counting partially sectioned cells by including only cells with clearly identifiable nuclei within a single focal plane.

To analyze the number of spiral ganglion neurons (SGNs) in the Rosenthal's canal, as identified in Figure 1, we used the methodology proposed by Otte et al. [22] The total number of SGN was then multiplied by 10 (to account for cells in the transition between slides), and later corrected by a predefined factor of 0.9 [22]. For the Scarpa's ganglion neurons (ScGNs) in vestibular nerve count, it was obtained using the methodology proposed by Richter [23] To avoid overestimation of data, we used the correction factor of 0.88 as described by Abercrombie [24] then the number of cells was also multiplied by 10 to account for unstained sections. The computer‐assisted labeling was used exclusively for the quantification of SGN and ScGN, and not for cochlear hair cell counts. For neuronal quantification, each neuron cell was identified based on the presence of a clearly defined nucleus and surrounding cell membrane. Once identified, each neuron was manually marked with a single digital “x” using the software interface. This approach ensured that every counted neuron was labeled only once, thereby minimizing the risk of double counting or inadvertent omission during the counting process. This method represents an improvement over traditional visual tally‐based counting, as it provides a persistent visual record of each identified cell within the image field and allows verification during review of the counts. A single observer (M.P.T.) performed all cell counts under the supervision of a senior researcher (R.C.M.).

For the statistical analysis, ANOVA was used to compare means between the three different groups. All statistical tests were performed using SPSS 29.0 and JASP 0.95.4. A *p* value of less than 0.05 was considered statistically significant.

## 3 Results

A total of 36 TBs met the inclusion criteria: 21 in the otogenic group, 15 in the meningogenic group, and 16 in the control group. The mean age was 11.56 years (standard deviation [SD] ±21.05) in the otogenic group and 36.40 years (±29.49) in the meningogenic group, representing a statistically significant difference (*p* = 0.005). Male predominance was observed in both groups (90% otogenic, 73% meningogenic). Demographic characteristics and statistical comparisons are summarized in Tables 1 and 2, respectively.

**TABLE 1:** Demographics (±SD) of each group and the differences between them.

Groups	Control	Otogenic	Meningogenic	Difference (*p*)		
Number of cases	16	21	15	Otogenic vs. meningogenic	Otogenic vs. control	Meningogenic vs. control
Sex (male:female)	87.5%	90%	73%			
Age (years)	22.05 (±15)	11.56 (±21)	36.40 (±29)	**0.005** *	0.341	0.184
Postmortem time (h)	8.73 (±4.5)	13.15 (±7.9)	14.81 (±10.7)	0.85	0.137	0.315

Abbreviation: ±SD, standard deviation.

* A *p* value of less than 0.05 was considered statistically significant indicated in bold.

**TABLE 2:** Means (±SD) of histopathological findings of each group and the differences between them.

Groups	Control	Otogenic	Meningogenic	Difference (*p*)		
				Otogenic vs. meningogenic	Otogenic vs. control	Meningogenic vs. control
Loss of OHC (%)	6 (±4)	60 (±30)	34.5 (±28)	**0.01** *	**< 0.001** *	**0.008** *
Loss of IHC (%)	5 (±1)	39.8 (±38)	13.7 (±23)	**0.019** *	**< 0.001** *	**0.382**
Number of SGN	11174.62 (±1632)	8335.28 (±3608)	8233.80 (±1941)	0.993	**0.007** *	**0.01** *
Number of ScGN	4933.50 (±1416)	2531.88 (±1294)	2214.66 (±1285)	0.762	**< 0.001** *	**< 0.001** *

Abbreviations: IHC, inner hair cells; OHC, outer hair cells; ScGN, scarpa's ganglion neurons; ±SD, standard deviation; SGN, spiral ganglion neurons.

* A *p* value of less than 0.05 was considered statistically significant indicated in bold.

### 3.1 OHCs

The otogenic group demonstrated a mean OHC loss of 60% (±30), significantly greater than the meningogenic group at 34.5% (±28; *p* = 0.01). Both groups exhibited significantly greater OHC loss compared to the control group (otogenic: *p* < 0.001; meningogenic: *p* = 0.008).

### 3.2 IHCs

**FIGURE 2:**
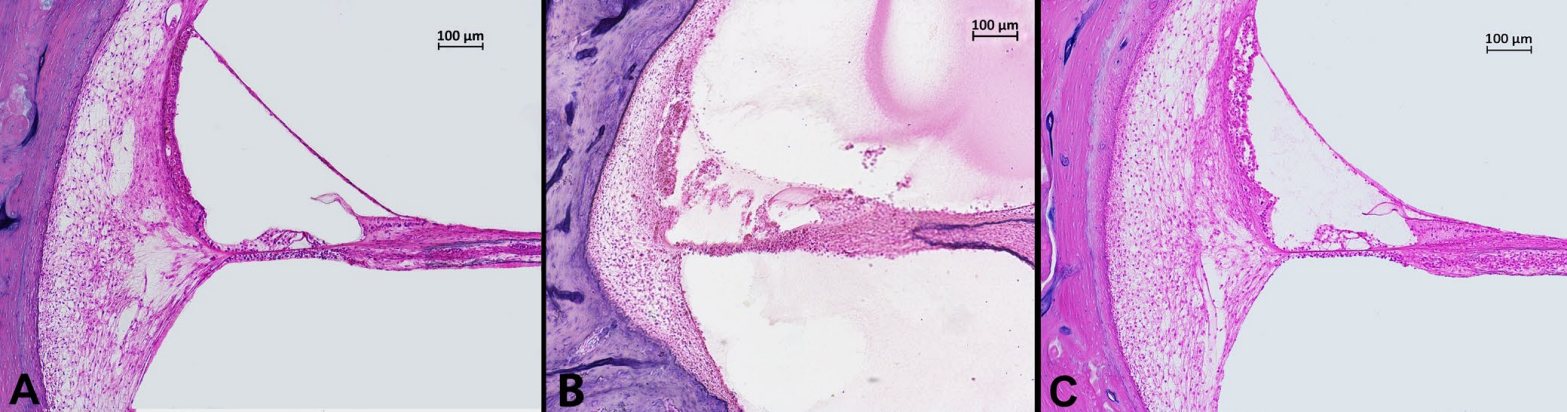
Representation of organ of corti and cochlear hair cells in each group. (A) Control group, exhibiting complete preservation of cochlear hair cells (hematoxylin–eosin, original magnification ×10). (B) Otogenic group, histopathologic findings include presence of inflammatory cells, sero‐purulent effusion, complete destruction of the organ of corti, complete loss of cochlear hair cells, inflammatory changes of the stria vascularis and spiral ligament, collapse of Reissner's membrane (hematoxylin–eosin, original magnification ×10). (C) Meningogenic group, histopathologic findings include partial loss of outer hair cells and presence of inner hair cell. (hematoxylin–eosin, original magnification ×10).

Mean IHC loss was 39.8% (±38.1) in the otogenic group and 13.7% (±23.5) in the meningogenic group. The otogenic group showed significantly greater IHC loss compared to both meningogenic (*p* = 0.019) and control groups (*p* < 0.001). No significant difference was observed between meningogenic and control groups (*p* = 0.382). Cochlear hair cell loss patterns across groups are illustrated in Figure 2.

### 3.3 SGNs

**FIGURE 3:**
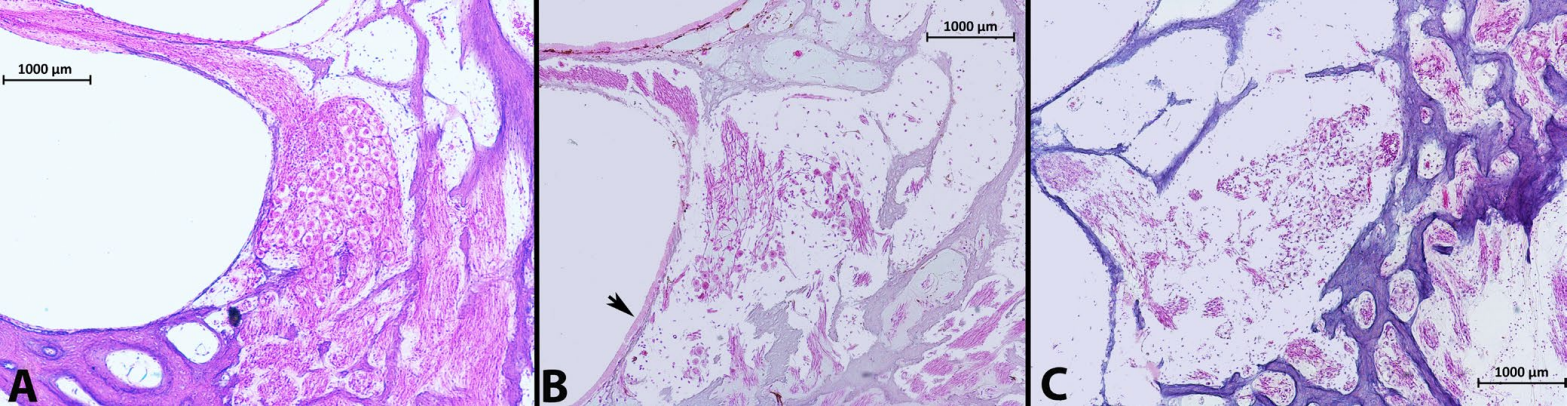
Representation of spiral ganglion neurons (SGN) in each group. (A) Control group (hematoxylin–eosin, original magnification ×10); (B) Otogenic group, histopathologic findings include SGN loss and the presence of purulent effusion (arrow) (hematoxylin–eosin, original magnification ×10). (C) Meningogenic group, exhibiting SGN loss (hematoxylin–eosin, original magnification ×10).

Mean SGN counts were 8335.28 cells (±3608.94) in the otogenic group and 8233.80 cells (±1941.14) in the meningogenic group. Both groups demonstrated significantly reduced SGN counts compared to the control group (otogenic: *p* = 0.007; meningogenic: *p* = 0.01), with no significant difference between the two pathological groups (*p* = 0.993). SGN distribution across groups is shown in Figure 3.

### 3.4 ScGNs

**FIGURE 4:**
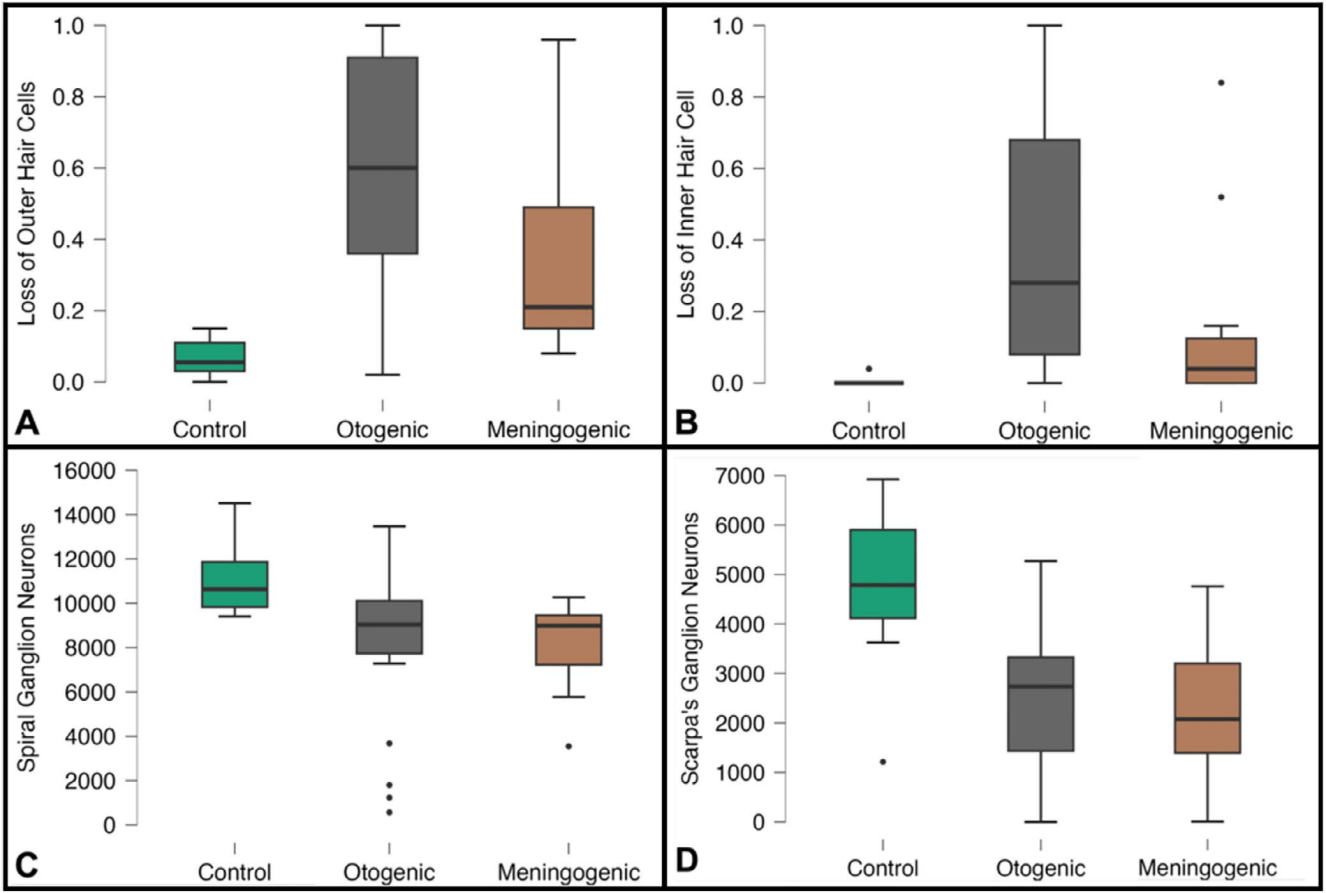
Boxplot of each statistical analysis. (A) Loss of outer hair cells. (B) Loss of inner hair cell. (C) Spiral ganglion neurons. (D) Scarpa's ganglion neurons.

Mean ScGN counts were 2531.88 cells (±1294.16) in the otogenic group and 2214.66 cells (±1285.97) in the meningogenic group. Both pathological groups showed significantly fewer ScGNs compared to the control group (both *p* < 0.001), with no significant difference between otogenic and meningogenic groups (*p* = 0.762). The statistical comparison of each group results is represented in Figure 4.

## 4 Discussion

Meningitis is a known cause of cochlear and vestibular cell loss. There is no previous study comparing the number of those cells in each route of meningitis. Our findings highlight distinct patterns of injury across the two groups, suggesting that the otogenic route displays greater cochlear cell loss in comparison to the meningogenic route. In our study, OHC loss was observed in both groups. However, the degree of loss was more pronounced in the otogenic group. Kaya et al. [25] reported loss of OHC in the labyrinthitis group in comparison to placebo, in both the lower and upper basal cochlear turns. We observed significant IHC loss only in the otogenic group. The meningogenic group showed a more severe loss in comparison to the control group, but it was not statistically significant. Kaya et al. [25] also demonstrated loss of IHC in the lower and upper basal cochlear turns in cases of labyrinthitis. However, they did not compare the different routes of meningitis. The reason for these findings may be attributed to the differential vulnerability of cochlear hair cells, as IHCs are generally more resistant to injury than OHCs. Additionally, it was demonstrated that otogenic cases display more extensive cochlear damage. Therefore, we should expect a less significant IHC loss in the meningogenic cases.

In our study, both otogenic and meningogenic groups showed statistically significant loss of SGN and ScGN. There was no difference between groups. Pauna et al. (2020) [11] reported ScGN loss in both the inferior and superior vestibular nerves in labyrinthitis cases; however, only the inferior vestibular nerve demonstrated statistically significant loss compared to age‐matched controls. SGN loss in cases of meningitis and labyrinthitis was also demonstrated in other studies [10, 11, 25, 26]. A sufficient number of SGN is considered to be an important factor for the success of cochlear implant surgery [22, 27, 28, 29]. Cochlear implantation has proven effective in post‐meningitic profound deafness [30, 31]; however, otologic surgeons should recognize that outcomes vary considerably. While SGN depletion has traditionally been implicated in suboptimal cochlear implant performance, our findings suggest that differential cochlear hair cell damage may also contribute to variable rehabilitation outcomes. Given the significantly greater hair cell loss observed in otogenic cases, this histopathological distinction provides a basis for investigating additional factors that may influence hearing rehabilitation success, including outcomes with alternative amplification devices.

Our reported SGN count of 11,175 in the control group with a mean age of 22 is lower than the expected total SGN populations reported by Otte et al. [22] and Makary et al. [32], which range from 30,000 to 40,000 for young adults. This discrepancy reflects a fundamental difference in counting methodology. We counted all SGN visible in five mid‐modiolar sections per specimen. In contrast, the referenced studies estimated the SGN population for the entire cochlea.

The significant age difference between groups (otogenic mean: 11.56 years; meningogenic mean: 36.40 years; *p* = 0.005) reflects well‐established epidemiological patterns. Otogenic meningitis predominantly affects younger patients due to higher rates of acute OM in pediatric populations [7, 33]. The majority of TB specimens in this study were obtained from individuals who died before 2000, predating widespread pneumococcal vaccination programs that have since reduced the incidence of both acute OM and bacterial meningitis in children. Notably, despite the younger age of the otogenic cohort—a factor typically associated with better cochlear preservation—this group demonstrated significantly greater hair cell loss than the older meningogenic group. This paradoxical finding strengthens the evidence that otogenic meningitis inflicts more severe cochlear damage independent of age‐related degeneration. The observed differences likely underestimate the true pathological impact of otogenic infection, as age‐adjusted analysis would theoretically reveal even greater disparities favoring more severe damage in the otogenic group. In two retrospective cohort studies [34, 35], HL in meningitis cases was associated with older age, pneumococcal infection, and concurrent OM. Meningococcal infection was associated with decreased odds of HL, and clinical severity of meningitis was not predictive for the development of HL.

The absence of documented audiovestibular history impeded our ability to correlate histopathologic findings with clinical manifestations. While the greater cochlear damage observed in otogenic cases suggests poorer auditory prognosis, prospective clinical studies with comprehensive audiological assessment are needed to definitively establish this relationship. Categorizing meningitis by route of infection—otogenic and meningogenic—provides a framework for understanding the pathogenesis of HL and anticipating differential auditory outcomes, though clinical validation of these histopathological findings remains essential. As central nervous system histopathology is not included in TB specimens, we were unable to analyze auditory cortex in order to investigate other possible causes of failed hearing rehabilitation. Notably, TB analysis cannot assess potential central auditory pathway involvement, which may also contribute to variable outcomes in some cases [36].

The analysis of only mid‐modiolar sections under light microscopy limited the complete and thorough assessment of quantitative cochlear and vestibular hair cells. Future studies should aim to completely count cochlear and vestibular cells and also include vestibular hair cells counting. Wu et al. [37] described a new technique to achieve a more accurate result for the extent of hair cell survival in TB. However, the high‐power differential interference contrast optics, which are necessary to apply this newer method, are currently unavailable in our laboratory. Despite this limitation, we used a widely accepted method across a large number of TB, allowing for direct comparison of our findings with those reported in the literature. A comparison between the histopathologic findings, audiovestibular symptoms, and testing results, as well as treatment regimen received, would be important to fully comprehend the pathogenesis of HL in meningitis, predict the severity of symptoms and sequelae, and also to predict therapy success rate.

## 5 Conclusion

Meningitis is associated with loss of cochlear hair cells, SGN, and ScGN. The otogenic route demonstrated a greater loss of both IHC and OHC in comparison with the meningogenic route.

## Funding

This project was funded by the National Institute on Deafness and Other Communication Disorders/National Institutes of Health (NIDCD/NIH) (U24 DC020851‐04), Minnesota Lions Hearing Foundation, and The National Council for Scientific and Technological Development (CNPq, Portuguese: Conselho Nacional de Desenvolvimento Científico e Tecnológico) (number 402014/2025‐1).

## Conflicts of Interest

The authors declare no conflicts of interest.

## Data Availability

The data that support the findings of this study are available from the corresponding author upon reasonable request.
